# Research on Single-Event Effect Hardening Method of Transverse Split-Gate Trench Metal-Oxide-Semiconductor Field-Effect Transistors

**DOI:** 10.3390/mi16040417

**Published:** 2025-03-31

**Authors:** Mengtian Bao, Ying Wang, Jianqun Yang, Xingji Li

**Affiliations:** 1 School of Electronic Information, Huzhou College, Huzhou 313000, China; 2 Information Science and Technology College, Dalian Maritime University, Dalian 116026, China; 3 National Key Laboratory of Materials Behavior and Evaluation Technology in Space Environment, Harbin Institute of Technology, Harbin 150080, China; yangjianqun@hit.edu.cn (J.Y.); lxj0218@hit.edu.cn (X.L.)

**Keywords:** power MOSFET, single-event effect, SEB hardening, process optimization

## Abstract

In this work, the single-event burnout (SEB) effect and degradation behaviors induced by heavy-ion irradiation are investigated in a 120 V-rated transverse split-gate trench (TSGT) power metal-oxide-semiconductor field-effect transistor (MOSFET). Bismuth heavy-ions are used to conduct heavy-ion irradiation tests. The experimental results show that the SEB failure threshold voltage (*V*_SEB_) of the tested sample is 72 V, which only accounts for 52.6% of the actual breakdown voltage of the device. The *V*_SEB_ value decreased with the increase in the flux. The simulation results show that the local “hot spot” formed after the incident heavy ion is an important reason for the drain current degradation of TSGT MOSFETs. To improve the single-event effect tolerance of TSGT MOSFETs, an SEB hardening method based on process optimization is proposed in this paper, which does not require additional customized epitaxial wafers. The simulation results show that, after SEB hardening, the *V*_SEB_ is increased to 115 V, which accounts for 89.1% of the breakdown voltage.

## 1. Introduction

Owing to their ultra-low power consumption and fast switching performance, split-gate trench (SGT) power metal-oxide-semiconductor field-effect transistors (MOSFETs) have been developed rapidly in the field of medium–low voltage control and conversion in aerospace systems [1,2,3]. However, conventional SGT MOSFET devices are sensitive to heavy-ion irradiation and are prone to single-event effects, resulting in the degradation of device performance and even catastrophic failures during on-orbit operation such as single-event burnout (SEB) [4,5,6].

To improve the SEB tolerance of power MOSFETs, many hardening studies have been carried out using experiments and simulations [7,8,9,10,11]. In recent years especially, the method of introducing an N-type buffer layer between the epitaxial layer and the substrate has been widely studied [12,13]. This hardening method can significantly increase the breakdown threshold voltage (*V*_SEB_) of a device [14,15]. However, it should be noted that in the actual manufacturing process of SGT MOSFET devices, the fabrication of the buffer layer is not included in the conventional process flow and requires additional customized epitaxial wafer implementation, and the difficulty and cost of customization increase with the thickness and concentration of the buffer layer.

In this work, an SEB hardening method based on process optimization is proposed, which does not require additional customized epitaxial wafers. We first tested the base breakdown voltage, transfer characteristics, and single-event effect of the TSGT MOSFET samples with a rated voltage of 120 V and analyzed the SEB performance of the samples to find the *V*_SEB_ value of the devices. Next, based on the test results, the samples under test are modeled using SILVACO TCAD simulator to simulate the above properties. On this basis, the performance of the hardened device is simulated using the process simulation method. The simulation results show that the process reinforcement method proposed in this paper can effectively improve the SEB tolerance of TSGT MOSFET devices.

## 2. Basic Device Performance and Irradiation Experiment Setup

**Figure 1:**
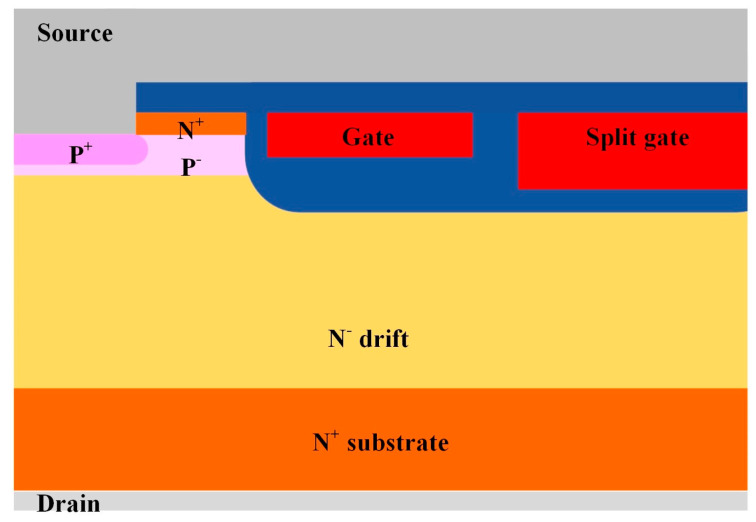
Half-cell cross-section of a 120 V-rated TSGT MOSFET.

Figure 1 illustrates the half-cell schematic cross-section of a 120 V-rated n-channel TSGT MOSFET. The thickness of the drift region is 9 μm, and the region has a doping concentration of 4.45 × 10^15^ cm^−3^. The thickness of the gate oxide layer is 60 nm. The length of the P-body region is about 0.57 μm. For enhancing the SEB performance, two important process steps are introduced: (1) a deep etch thickness of 0.4 μm is set in the trench source contact, and (2) an extension of the P+ base under the trench source contact is formed.

Before the irradiation experiments, we tested the transfer and breakdown characteristics of the samples to ensure the smooth progress of the subsequent irradiation experiments. Furthermore, the experiment results could also provide a reference for the subsequent theoretical analysis and modeling of irradiation hardening. To distinguish the other structures mentioned later, we call the 120 V-rated TSGT MOSFET the original TSGT.

**Figure 2:**
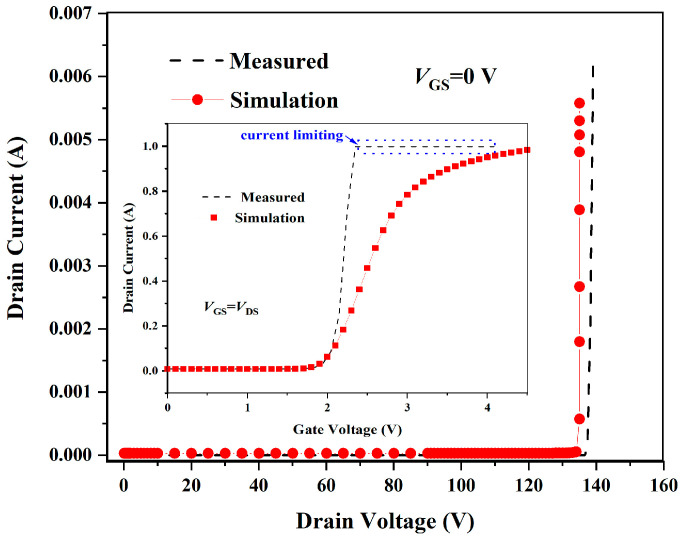
Comparison of transfer characteristics (inset of Figure 2) and breakdown characteristics of the 120 V-rated TSGT MOSFET obtained via measurements and simulations.

SILVACO ATHENA and ATLAS simulators are used to model the structure and simulate the performance of the original TSGT, respectively. On this basis, through the optimization of parameters and models, the error between the measured values and simulation results is reduced, and the purpose of calibrating the simulation program of the proposed TSGT MOSFET is to ensure the accuracy of subsequent simulations. The proposed TSGT MOSFET is simulated by a standard process flow. As shown in Figure 2, the experimental results show that the threshold voltage (*V*_TH_) of the sample under test is about 1.9 V under *V*_GS_ = *V*_DS_ conditions, which is in accordance with the reference range given in the datasheet. The *I*-*V* characterization test results of the samples under the condition of *V*_GS_ = 0 V show that the actual breakdown voltage (*BV*) of the device is 137 V. The simulation results show that the proposed structure has a *V*_TH_ of 2.0 V and a *BV* of 135 V. Compared with the measurement results, the error of the threshold voltage Δ*V*_TH_ is 5.26%, and the error of breakdown voltage Δ*BV* is 1.46%. As shown in Figure 2, the error between all the simulation results and the test data is within the allowable range; both have a good fit.

The single-event effect experiments were conducted with Bismuth heavy-ions using the Space Environment Ground Simulation Device (Harbin). A Bi ion beam with a linear energy transfer (LET) of 99.8 MeV·cm^2^/mg was vertically incident on the samples. The plastic packaging material was removed from samples before the experiment. During the irradiation, samples under test were biased at a fixed drain voltage. The test system provides overload protection to limit the drain and gate currents. The drain and gate currents were monitored with three different fluxes: 5.18 × 10^3^ ions·cm^−2^·s^−1^, 8.0 × 10^3^ ions·cm^−2^·s^−1^ and 1.0 × 10^4^ ions·cm^−2^·s^−1^, respectively. The performance of the original TSGT MOSFET against heavy-ion irradiation was calculated using mobility models, carrier statistic models, Selberherr’s impact ionization model, and Shockley–Read–Hall (SRH) and Auger recombination models. The SEB effect will cause a change in the internal junction temperature of the tested sample. Therefore, the electrothermal model is also used for device performance simulation.

## 3. SEB Experiment Results and Analysis

### 3.1. Drain Current and SEB Threshold

**Figure 3:**
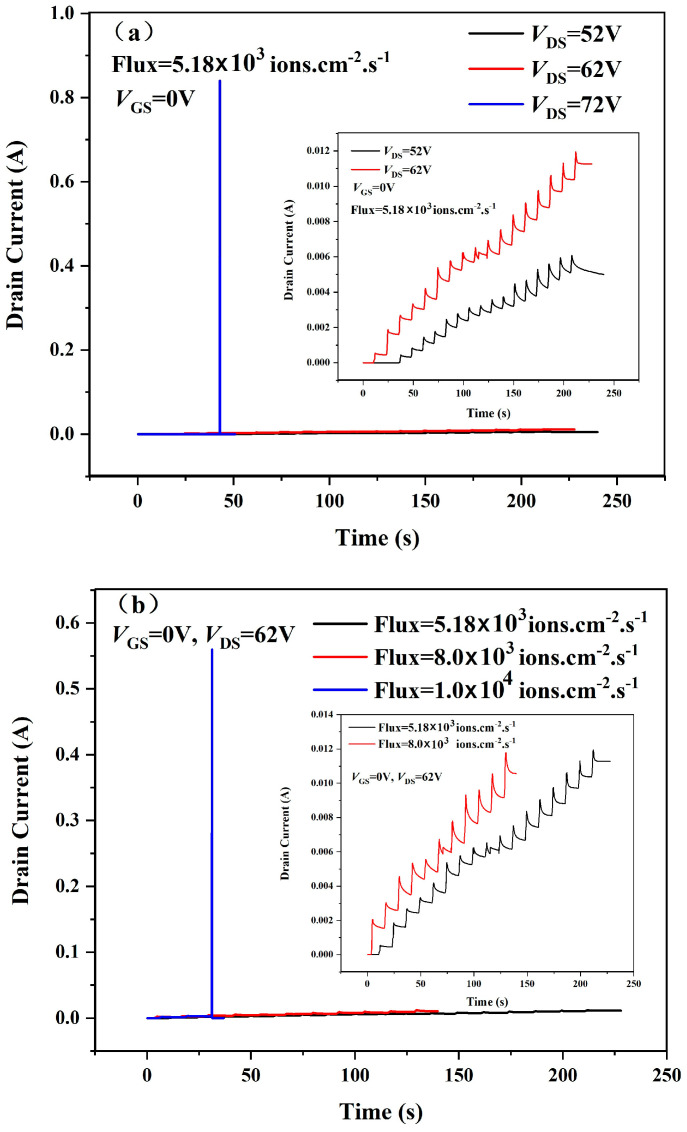
Measured drain leakage degradation curves for the 120 V-rated TSGT MOSFET. (**a**) Measurement of the SEB threshold voltage for the 120 V-rated TSGT MOSFET. (**b**) Effects of different flux conditions on the drain leakage current.

Figure 3a shows the changes in instantaneous drain current with time for 120 V-rated samples under different *V*_DS_ conditions when flux = 5.18 × 10^3^ ions·cm^−2^·s^−1^ and *V*_GS_ = 0 V. To ensure that the sample is completely damaged after irradiation, the SEB damage judgment condition of the sample is set as *I*_D_ or *I*_G_ reaching the overcurrent protection value of the test system. The *V*_DS_ value corresponding to SEB damage was defined as the SEB failure threshold voltage (*V*_SEB_), which was used to characterize the SEB properties of the sample. As shown in the insert of Figure 3a, significant drain current degradation occurred in the sample under test when *V*_DS_ = 52 V and 62 V, respectively. The experimental results show that the overcurrent protection of the hardware test system is triggered by the heavy-ion incidence when *V*_DS_ = 72 V, so the *V*_SEB_ with the flux = 5.18 × 10^3^ ions·cm^−2^·s^−1^ of the 120 V-rated TSGT MOSFET sample is 72 V.

Figure 3b shows the changes in instantaneous drain current with time for 120 V-rated samples under different flux conditions when *V*_DS_ = 62 V and *V*_GS_ = 0 V. From the experimental results, when the bias conditions are certain, the heavy-ion beam flux is larger, and the drain current value of the sample under test increases subsequently. This ultimately leads to a significant decrease in the *V*_SEB_ value of the tested samples. This is due to the increase in the number of heavy ions incident on the device per unit time as the flux increases and the increase in the number of electron–hole pairs induced by the heavy ions, which in turn leads to an increase in the impact ionization rate at critical locations within the device. The mapping of the above microphysical processes onto the device performance is what leads to a significant increase in device drain current. In Figure 3b, when flux = 1.0 × 10^4^ ions·cm^−2^·s^−1^, the *V*_SEB_ of the 120 V-rated TSGT MOSFET is 62 V.

### 3.2. SEB Simulation and Analysis

**Figure 4:**
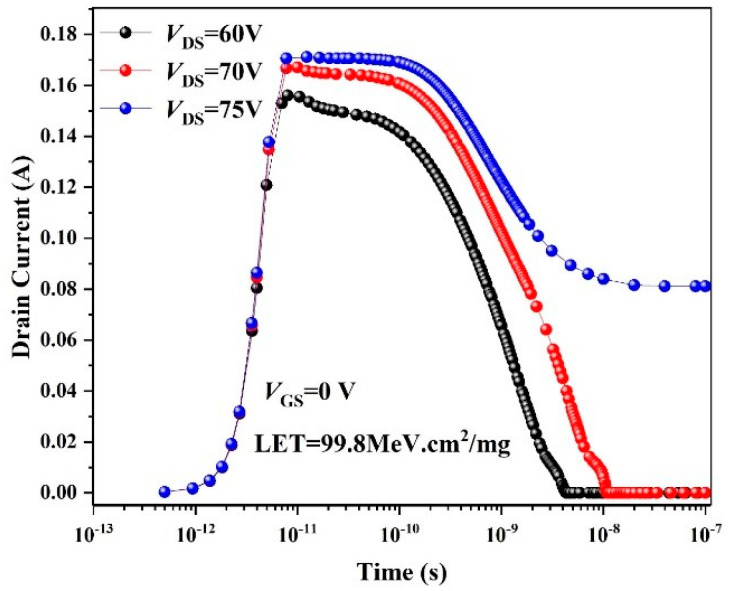
Simulated drain leakage current for the 120 V-rated TSGT MOSFET after ion striking at different drain voltages. Ion LET = 99.8 MeV·cm^2^/mg and *V*_GS_ = 0 V.

The simulation results of the SEB performance of the original TSGT structure against heavy-ion irradiation are shown in Figure 4. Transient drain current over time is simulated, in which the conditions involve a heavy ion with an LET of 0.998 pC/μm (1 pC/μm = 100 MeV·cm^2^/mg in silicon) [16] striking the channel location to penetrate through the structure. Consequently, when *V*_DS_ = 75 V, the drain current appears as a long tail phenomenon that does not return to 0 A. The current is maintained near 0.08 A, which is far beyond the range of normal drain leakage current, indicating that the device has irreversible damage. Therefore, the *V*_SEB_ of the simulated TSGT is 75 V, which is very close to the measured value shown in Figure 3a, and the error of Δ*V*_SEB_ is 4.17%.

**Figure 5:**
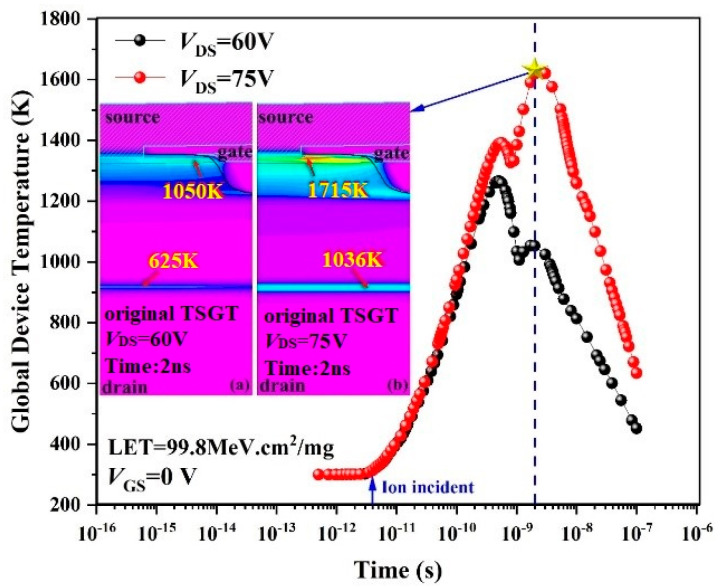
Trend of global device temperature for the 120 V-rated TSGT MOSFET after heavy-ion incidence. Maximum temperature distributions of the simulated structure for 2 ns after a heavy-ion strike at (**a**) *V*_DS_ = 60 V and (**b**) *V*_DS_ = 75 V.

To further investigate the thermal effects due to heavy-ion radiation on the TSGT MOSFET device, the variation in the maximum internal temperature of the device with time under the same conditions shown in Figure 4 is shown in Figure 5.

As shown in Figure 5, when heavy ions are incident at *t* = 4 ps, with the increase in electron–hole pairs, the internal lattice temperature of the device increases, and a hot spot is generated near the channel on the surface of the device. When *V*_DS_ = 60 V, the highest lattice temperature occurs at *t* = 0.8 ns, and the peak temperature is about 1300 K. As *V*_DS_ increases, the internal electric field of the device is improved, and the impact ionization rate of carrier is more intense, and the lattice temperature increases significantly. At *t* > 1 ns, avalanche breakdown occurs inside the device, and heat accumulates at critical locations, eventually leading to the N^+^/P^−^ junction temperature exceeding the melting point of Si material at 1700 K. As shown in the inset of Figure 5, the maximum lattice temperature is 1715 K when *V*_DS_ = *V*_SEB_, which triggers the SEB effect. This result further confirms the variation in drain current shown in Figure 4.

## 4. SEB Hardening Methods

### 4.1. Substrate Hardening Process on SEB

Although the reinforcement against the SEB effect has been considered in the simulation modeling process in this paper as shown in the Section 2, the resistance to SEB of the 120 V-rated TSGT is not satisfactory as observed from the test data and simulation results. According to the simulation results, the *V*_SEB_ of the proposed device is 75 V, which is only 55.6% of the device breakdown voltage. To enhance the SEB performance of a 120 V-rated TSGT MOSFET, the introduction of a buffer layer between the device drift region and the substrate has become a recognized and effective reinforcement method [17,18]. However, in the actual fabrication of the device, buffer layer fabrication is not included in the standard process flow and needs to be realized by additional customized epitaxial wafers, and the cost of customization increases with the thickness and concentration of the buffer layer.

To solve the problem of process compatibility of the buffer layer in the production process of TSGT MOSFET devices, we propose a process method using high-temperature annealing to make the N-type impurities in the substrate back-propel into the drift region to form a buffer layer.

**Figure 6:**
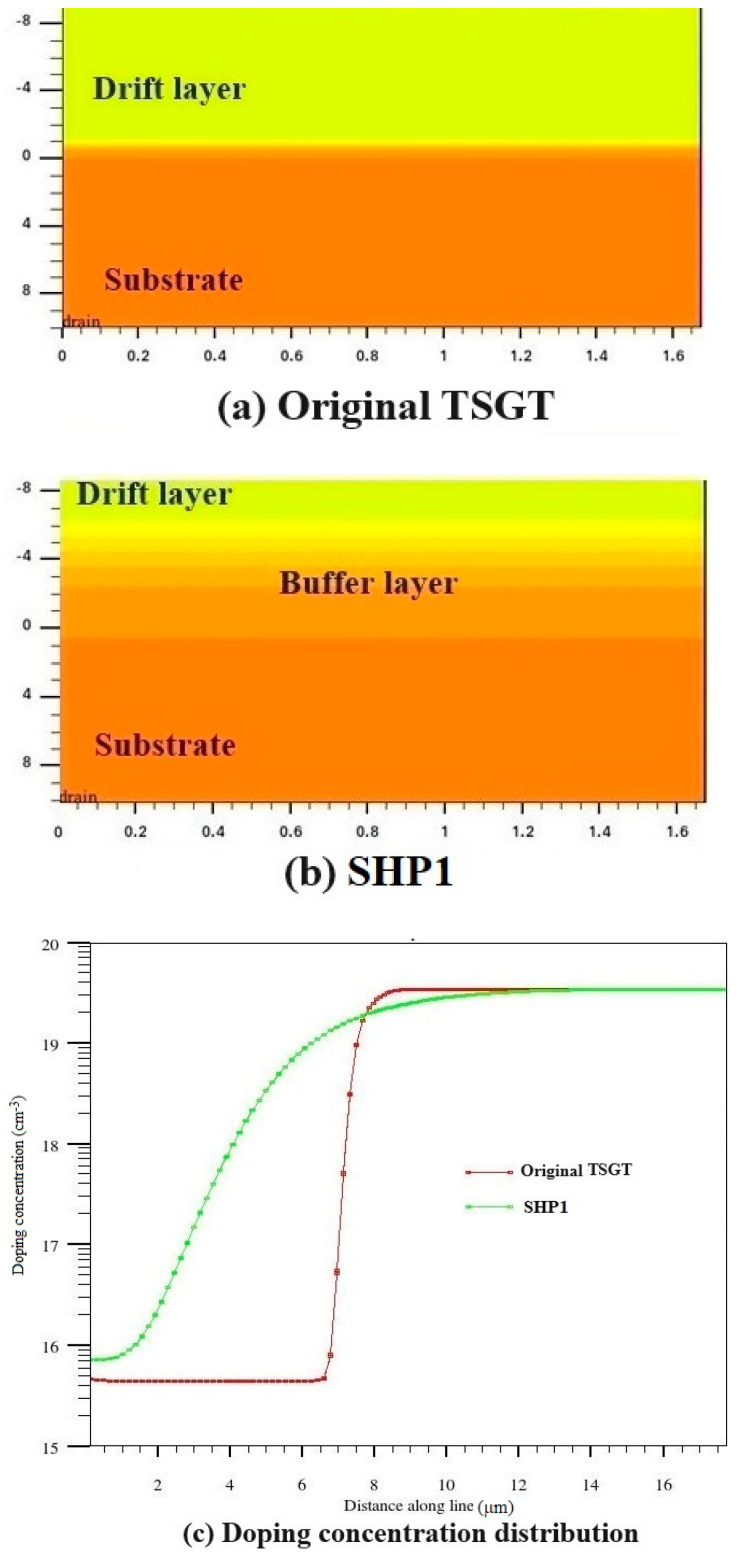
The structure distribution of (**a**) the original TSGT and (**b**) the hardening TSGT with the buffer layer fabricated using the optimal scheme SHP1 and (**c**) the doping concentration distribution for both devices.

Based on a large number of simulation experiments, we found a buffer layer formation method with the most ideal SEB hardening effect, and its key process parameters are temperature = 1150 °C and time = 2200 min, which is named as substrate hardening process 1 (SHP1). The structure and doping concentration distribution of the original TSGT and the hardening TSGT with a buffer layer fabricated using the optimal scheme, SHP1, are shown in Figure 6. As can be seen from Figure 6, the effective thickness of the buffer layer is about 5 μm. The maximum electric field could decrease and shift from the N^−^-N^+^ junction to the N^+^-N^++^ interface by the buffer layer introduced, which allows the device to support a much higher electric field [19].

**Figure 7:**
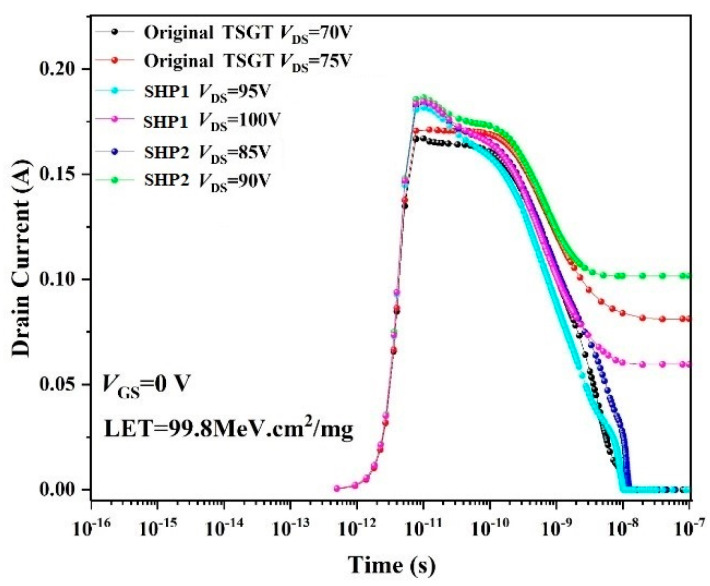
The trend of change in drain leakage current with time and the influence of reinforcement methods on *V*_SEB_ after incident heavy ions.

We similarly tried to optimize the substrate resistivity, annealing temperature, time, and other influencing factors, such as the hardening TSGT with a buffer layer fabricated using plan 2, whose time = 2000 min. The reinforcement structure obtained by using this unoptimized process named as SHP 2. However, the SEB hardening effect is not satisfactory. The simulation results of the SEB effect of different structures are given in Figure 7.

To more clearly show the influence of reinforcement process optimization on the radiation hardening of the device, Figure 7 shows the change in the drain current of different devices with time after heavy-ion irradiation. As shown in Figure 7, the SEB failure threshold voltage of the TSGT MOSFET device is increased from 75 V to 100 V using the buffer layer process optimization method (SHP1). The buffer layer formed by process optimization effectively reduces the effect of incident heavy ions on device drain current and significantly improves the *V*_SEB_ of TSGT MOSFET.

**Figure 8:**
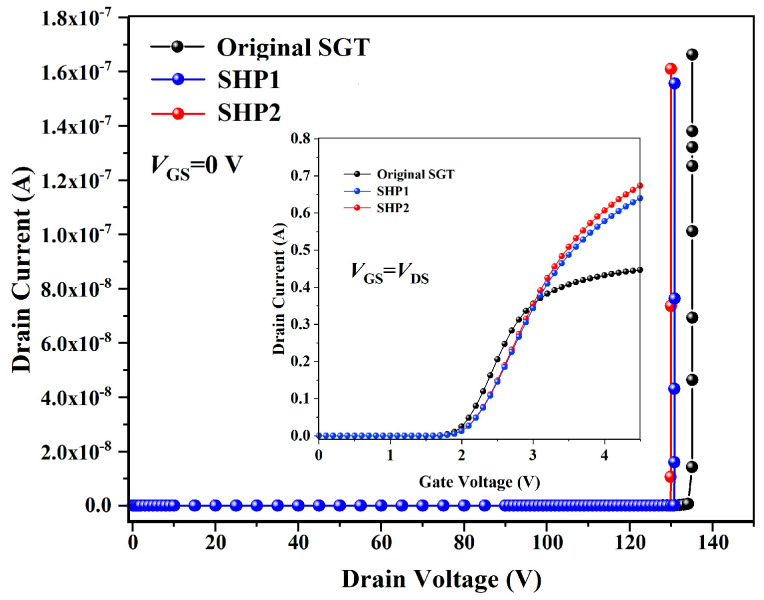
Comparison of transfer characteristics and breakdown characteristics of original TSGT, SHP1, and SHP2.

On the other hand, measuring the effectiveness of SEB hardening methods also requires consideration of the sacrifice of the key performance value of the reinforced structure. Figure 8 gives the variation in threshold voltage and breakdown voltage of the devices after using different reinforcement methods. As can been seen from Figure 8, the threshold voltages of the devices are basically unchanged after adopting the buffer layer reinforcement method, and the breakdown voltages decrease to 130 V, which is an acceptable result.

### 4.2. Active Area Hardening Process on SEB

**Figure 9:**
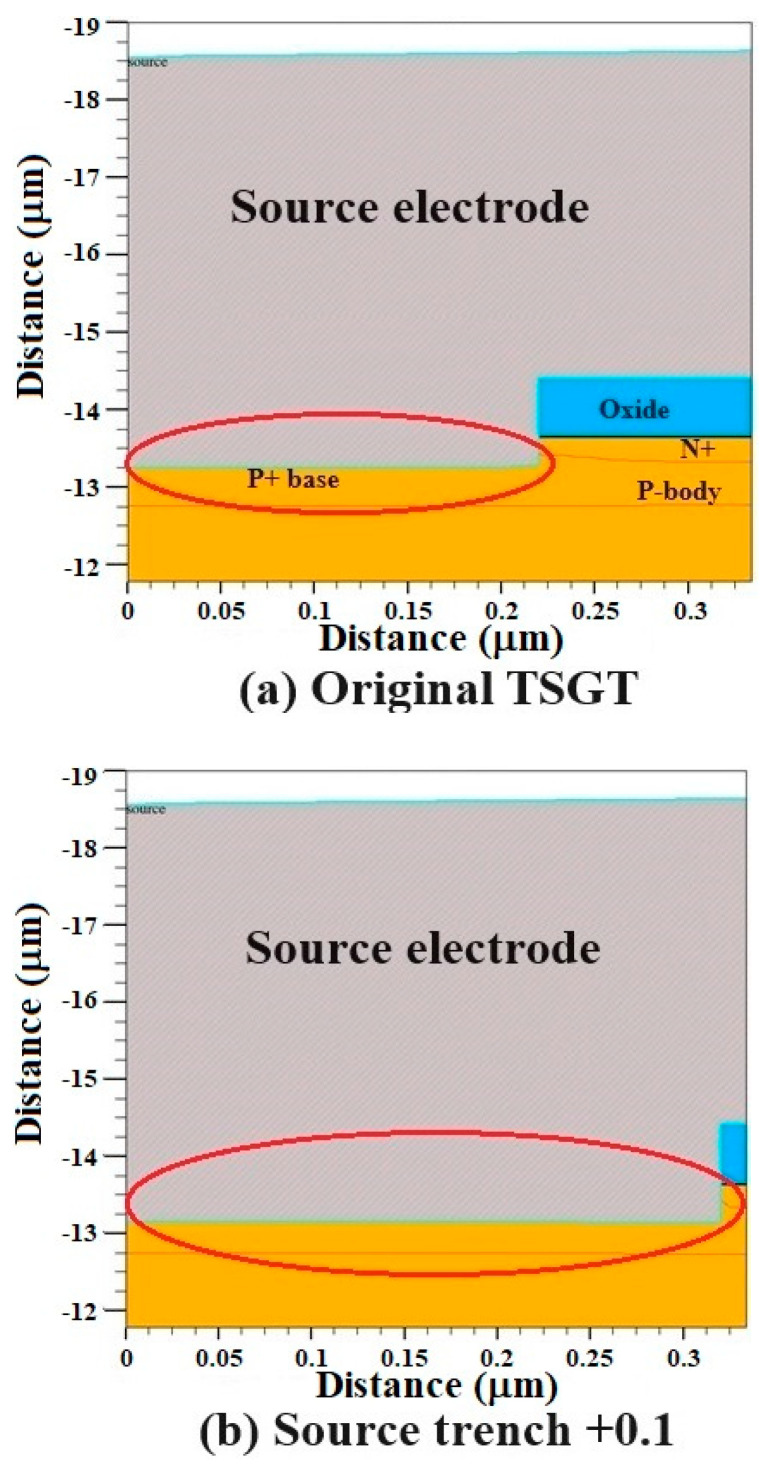
The TSGT reinforced structure is obtained after the source trench is extended by 0.1 μm along the X and Y axes, respectively.

**Figure 10:**
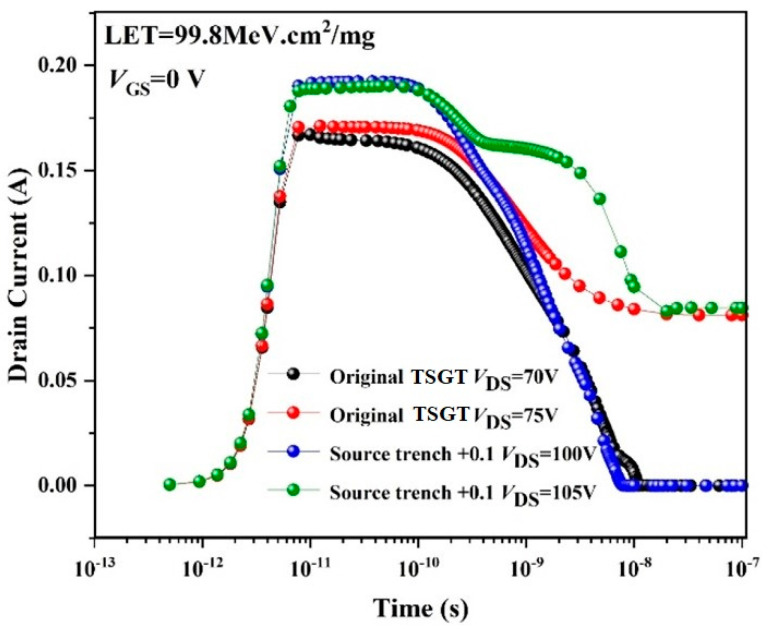
Comparison of *V*_SEB_ between original TSGT and hardened structure by source trench bi-directional expansion after heavy-ion striking.

Another hardening method that can effectively improve the SEB tolerance of 120 V-rated TSGT MOSFET is to optimize the source trench structure. As shown in Figure 9, based on the original TSGT structure, the source trench is extended horizontally and vertically, respectively, and the extension value is 0.1 μm, while the other structural parameters remain unchanged. We use the “source trench +0.1” to indicate the above reinforced structure. The SEB failure threshold voltage of the hardened device can reach to 105 V, as shown in Figure 10. Compared to the original TSGT structure, when *V*_DS_ is large enough, the gate oxide electric field strength and range of the source trench extended structure increases significantly, and a wider range of electric field distribution exists near the N^−^-N^++^ junction between the drift region and the substrate. As a result, the electric field inside the device takes longer to rebuild, and the *I*_D_ curve exhibits a platform phenomenon before the drain current reaches saturation when *V*_DS_ = 105 V. Extending the source channel can effectively reduce the area of the parasitic BJT region and increase the on-state threshold of the parasitic transistor, thus effectively improving the *V*_SEB_ of the device.

**Figure 11:**
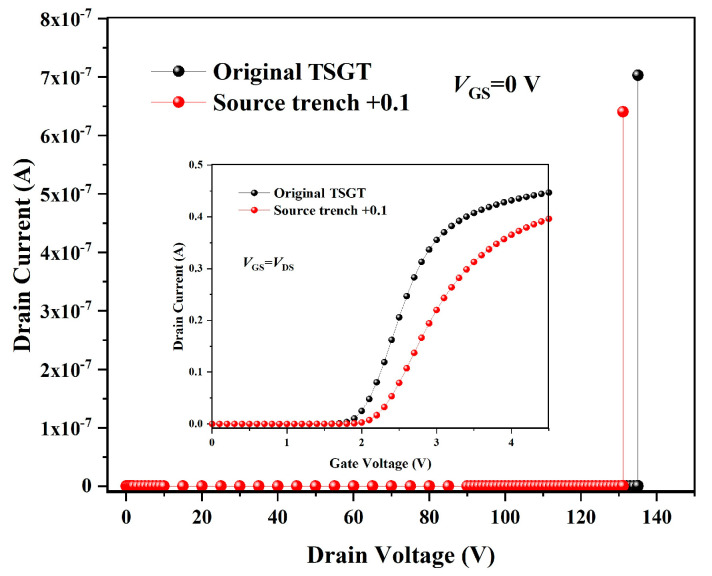
Comparison of the transfer and breakdown characteristics of the original TSGT and source trench bi-directional expansion structure.

The simulation results show that the source trench bi-directional expansion structure can improve the *V*_SEB_ value of the original TSGT device more effectively than the buffer layer method; however, under the same *BV* conditions, the threshold voltage of the source trench bi-directional expansion structure is larger (*V*_TH_ = 2.2 V, shown in Figure 11). The source trench extension is equivalent to increasing the doping concentration in the channel region. Therefore, the threshold voltage of the source trench extension reinforcement structure is large, which has a greater impact on the comprehensive performance of the device.

### 4.3. Composite Hardening Method

**Figure 12:**
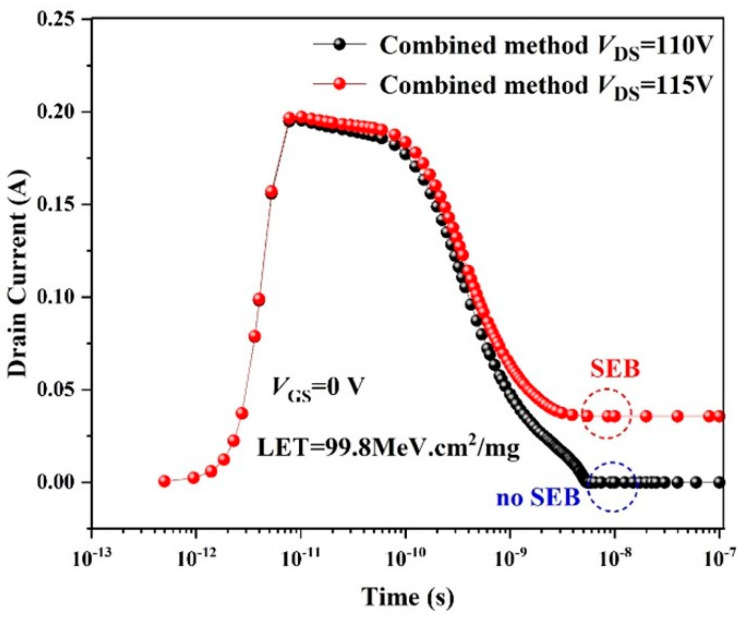
Trend of the instantaneous drain leakage current of the combined structure after heavy-ion incidence at *V*_DS_ = 110 V and *V*_DS_ = 115 V. Ion LET = 99.8 MeV·cm^2^/mg and *V*_GS_ = 0 V.

Summarizing the results of the previous subsections, to further improve the SEB tolerance of a 120 V-rated TSGT MOSFET, this paper combines the buffer layer hardening method with the source trench bi-directional expansion structure. This work optimizes the original TSGT simultaneously from both the front and back of the device. As shown in Figure 12, the *V*_SEB_ of the device can be increased to 115 V with the composite hardening method, which accounts for 89.1% of the actual breakdown voltage. Compared with the original TSGT, the SEB tolerance of the composite hardening structure is improved by 53.3%.

**Figure 13:**
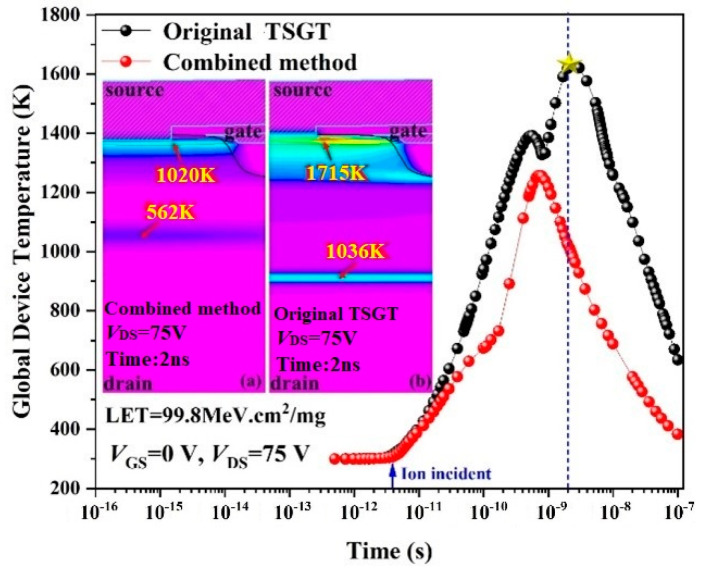
Trend of the lattice temperature of the 120 V-rated TSGT MOSFET and hardened structure by the combined method after heavy-ion incidence. Maximum temperature distributions of the (**a**) hardened structure by combined method and (**b**) the original TSGT for 2 ns after a heavy-ion strike at *V*_DS_ = 75 V.

Figure 13 shows the lattice temperature changes in the combined TSGT MOSFET and the original TSGT MOSFET under the same conditions. As can be seen from the figure, the simultaneous SEB hardened the TSGT MOSFET substrate, and the active area can effectively alleviate the heat accumulation on the surface and inside of the device. By using the composite hardening method, the lattice temperature at the channel of the device drops to 1020 K, the hot spot between the drift region and the substrate shifts to the N^−^-N^+^ junction between the drift region and the buffer layer, and the temperature drops to 562 K. Therefore, the SEB tolerance of the hardened TSGT MOSFET device is greatly improved.

Although the composite hardening method substantially increases the SEB failure threshold voltage of the TSGT MOSFET device, its impact on the fundamental performance of the device is also noteworthy. Table 1 gives the simulation results of the key performance of the original TSGT and the different hardened structures. The comparison shows that, although the device’s *BV* and *V*_TH_ are sacrificed to some extent, their values are within the metrics given in the datasheet of the 120 V-rated TSGT sample. Therefore, the SEB process hardening method for TSGT MOSFETs proposed in this paper is practical.

## 5. Conclusions

The SEB effect and hardening method in a 120 V-rated TSGT MOSFET are introduced in this work. The experimental results show that the SEB failure threshold voltage of the tested sample is 72 V. The trend of the device drain current is influenced by heat effects caused by heavy-ion radiation. To improve the single-event effect tolerance of TSGT MOSFET, an SEB hardening method based on process optimization is proposed. The approach consists of simultaneously optimizing the front-side and back-side processes of the device: (1) pushing the substrate impurities back into the drift region to form a buffer layer under high-temperature conditions and (2) extending the device source trench in the appropriate range of transverse and longitudinal extensions. The simulation results show that the base performance of the device is within the allowable range, and the *V*_SEB_ is increased from 75 V to 115 V, and the SEB tolerance performance of the device is significantly improved.

## Figures and Tables

**Table 1:** Comparison of key simulated properties of different structures.

	*BV* (V)	*V*_TH_ (V)	*R*_on_ (Ω)	*V*_SEB_ (V)
Original TSGT	135	2	1.2	75
SHP1	130	2.1	1.22	100
Source trench +0.1	130	2.2	1.6	105
Combined method	129	2.4	2.29	115

## Data Availability

The original contributions presented in the study are included in the article, further inquiries can be directed to the corresponding author.
